# Current Biosafety Considerations in Stem Cell Therapy 

**DOI:** 10.22074/cellj.2016.4324

**Published:** 2016-05-30

**Authors:** Masoumeh Mousavinejad, Peter W. Andrews, Elham Kargar Shoraki

**Affiliations:** 1 Centre for Stem Cell Biology (CSCB), Department of Biomedical Science, The University of Sheffield, Sheffield, UK; 2 Department of Biological Sciences, Faculty of Science, Tehran Kharazmi University, Tehran, Iran

**Keywords:** Safety, Tumorigenicity, Immunogenicity

## Abstract

Stem cells can be valuable model systems for drug discovery and modelling human diseases as well as to investigate cellular interactions and molecular events in the early stages of development. Controlling the differentiation of stem cells into specific germ layers provides a potential source of highly specialized cells for therapeutic applications. In recent years, finding individual properties of stem cells such as their ultimate self-renewal capacity and the generation of particular cell lines by differentiation under specific culture conditions underpins the development of regenerative therapies. These futures make stem cells a leading candidate to treat a wide range of diseases. Nevertheless, as with all novel treatments, safety issues are one of the barriers that should be overcome to guarantee the quality of a patient’s life after stem cell therapy. Many studies have pointed to a large gap in our knowledge about the therapeutic applications of these cells. This gap clearly shows the importance of biosafety concerns for the current status of cell-based therapies, even more than their therapeutic efficacy. Currently, scientists report that tumorigenicity and immunogenicity are the two most important associated cell-based therapy risks. In principle, intrinsic factors such as cell characteristics and extrinsic elements introduced by manufacturing of stem cells can result in tumor formation and immunological reactions after stem cell transplantation. Therapeutic research shows there are many biological questions regarding safety issues of stem cell clinical applications. Stem cell therapy is a rapidly advancing field that needs to focus more on finding a comprehensive technology for assessing risk. A variety of risk factors (from intrinsic to extrinsic) should be considered for safe clinical stem cell therapies.

Stem cells have the strong potential to differentiate into all three germ layers (endoderm, mesoderm, and ectoderm) and subsequently generate a broad range of cells such as cardiomyocytes, neurons, and hepatocytes (1). According to this potential, cell-based therapeutics aim to develop a set of techniques that replace damaged cells with healthy and proper functional ones derived from stem cells (2). Stem cell transplantation is still experimental and has a long process before it can be used as a clinical approach for humans. However, its promising treatments are indicated for numerous diseases and conditions such as cancers, neurodegenerative disorders and diabetes, in which the functions of specialized groups of cells have failed (3). Stem cells are a therapeutic promise not only for life threatening conditions, but also for chronic problems such as hearing or vision problems and even alopecia (4,5). 

There are two types of stem cells which can be used or have the potential to be used in stemcell-based therapies, multipotent [adult stem cells (ASCs)] (6) and pluripotent. The latter include embryonic stem cells (ESCs) and induced pluripotent stem cells (IPSCs) (7). These cells can be harvested and transplanted into the patient via two main routes: allogeneic (donor-derived) stem cell transplantation of stem cells from a healthy donor, as adult or ESCs (Fig.1A), (8) or autologous (self-derived) stem cells that are derived from the patient’s body (Fig.1B) (9). 

**Fig.1:**
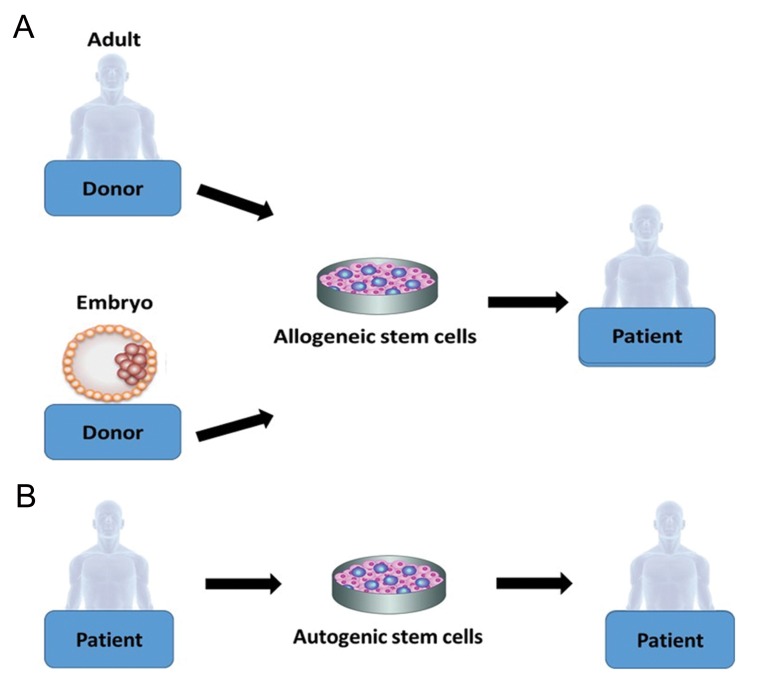
Harvesting stem cells for transplantation. A. Allogeneic and B. Autogenic stem cells are the two types of stem cell transplantations.

Despite rapid progress, stem cell therapy is surrounded by major difficulties that affect transplantation efficacy. Allogeneic transplantation can raise sharp ethical issues about human identity, especially when the original stem cells have been isolated from an embryo for further culture and manipulation. In addition, there is the risk of a high immune rejection (10). While autogenic stem cells avoid the ethical dilemma as well as graft rejection, this type of stem cell transplantation has a number of drawbacks. In autogenic stem cell therapy as treatment for cancer, there is the possibility of picking up cancer cells along with stem cells (11,12). 

Regardless of the stem cell source (ASCs, ESCs or IPSCs), stem cell therapy as with other treatments, has a number of side effects that patients’ experiences when they pass through the recovery stage. The effects of stem cell therapy after hematopoietic allogeneic transplantation, a common stem cell transplantation in humans, can be divided into two categories non-malignant and malignant (13). The non-malignant effects are inharmonious and can include ocular and pulmonary effects, liver complications, complications of bones and joints, dental effects, and puberty and gonadal failure (14). The malignant effects have serious harmful results such as leukemia, lymphoma, and suppression of the immune system (15). Recent researches have reported that significant tumor development and immune system failure are the two most serious reactions after stem cell transplantation (7,16). As reported, mesenchymal stem cells (MSCs) have been used to treat acute and chronic liver damage in an animal model. These cells had proper differentiation into hepatocyte-like cells at the injury site, however the side effects from the MSCs infusion included a fibrotic process and uncertain long-term efficacy (17). Alderazi et al. (18) examined the case study of a 17-year-old girl who underwent stem cell therapy nine months after she developed symptoms of multiple sclerosis. The patient had complaints of headaches. After a brain biopsy and magnetic resonance imaging (MRI), she was determined to have catastrophic demyelinating encephalomyelitis due to the immune system attack on her brain. In a long-term study for posttransplantation efficacy, researchers identified that from 3337 female allogeneic transplant patients, 52 developed breast cancer as a long-term effect. Hence the risk of breast cancer increased after allogeneic hematopoietic cell therapy (19). 

Ultimately, according to such research, major concerns exist regarding the actual risk of tumorigenesis and immunosuppression, both immediately and long-term after stem cell transplantation. Several researches have indicated that these two effects are highly associated with the stem cell therapy procedures (3). On the other hand, a number of papers discussed the link between the molecular basis of pluripotency and tumorigenicity (1,7). In this paper, we have intended to discuss some current biosafety issues and associated risk factors of stem cell therapy. Details on safety issues of tumorigenicity and immunogenicity will be addressed. One of the useful characteristics of undifferentiated stem cells is the capacity for rapid growth from a tightly packed colony with a low rate of spontaneous differentiation. However, uncontrolled growth of stem cells can result in tumor formation (20). Generally, researchers in regenerative medicine attempt to select and work with the stem cell lines that meet the above criteria. Stem cells with the above mentioned molecular characteristic can easily grow but at the same time such this characteristic enhances the chance for tumorigenicity by stem cell transplantation (21). Although it is unlikely that undifferentiated stem cells can directly be used for stem cell therapy, the ultimate growth potential raises the risk of tumor development (22). On the other hand, one of the initial steps in stem cell therapy is to develop desired cell types from undifferentiated cells before transplantation, which involves stem cell isolation followed by cultivation in appropriate culture medium. Isolation of stem cells from an organism (adult or embryo) and growing them in culture media are unavoidable steps in most cases of stem cell transplantation (3). In-depth researches have shown that a large number of mutations and karyotypic alterations can occur during *in vitro* cultivation of stem cells which enhances the tumorigenicity risk (23,24). 

The main reasons behind the high risk for tumor development by stem cell therapy are classified into two broad categories: genetic elements, which are referred to as intrinsic factors and the nature of stem cells, and epigenetic changes or extrinsic factors, which mainly occur during handling and manufacturing of stem cells in order to generate the desired cell type for transplantation (7). 

Recent study shows a shared molecular machinery between tumor and stem cells that indicates a link exists between tumorigenicity and pluripotency (25). The conserved gene networks between stem cells and tumor cells are implicated in a number of fundamental features such as rapid proliferation, uncoupling the DNA repair checkpoint, and high self-renewal capacity (1). The proto-oncogene is used to produce IPSCs such as the c-MYC transcription factor family (one of the important pluripotency genes); its overexpression can result in cancer in humans (20). Although it is possible to form IPSCs without or with lower levels of c-MYC gene reprogramming in order to have safer transplantation, omission of c-MYC can cause dramatic reduction of pluripotency (20,26,27). As a result, the time frame for expansion of stem cell colonies greatly extends, and mutations in the incubated cells in the culture medium will be inevitable (3). In addition to the *c-MYC* family, genes such as *NANOG, SOX2, KLF4* and *OCT4*, as core pluripotency master genes in ESCs, are closely associated with tumorigenicity. For example, *KLF4* suppresses *p53* in breast cancer whereas *SOX2* has been reported to promote cancer cell survival in lung cancer (3,28). Unfortunately greater pluripotency of stem cells increases the risk for tumor formation. 

Recent studies have reported that the oncogenic activity of stem cells is not only associated with undifferentiated cells. Therefore, differentiated stem cells used for stem cell therapy can reactive oncogenic properties such as resistance to apoptosis, lack of contact inhibition, and loss of *p53* (28,29). The dualistic natures of pluripotency genes show that stem cell therapy is faced with a large safety issue when used for clinical applications. 

Tumor development after stem cell transplantation is the undesirable effect that results from epigenetic changes during the main steps of the stem cell preparation, including stem cell isolation, cultivation, and injection into the patient at the appropriate dosage (26). Due to the extracellular and intracellular impacts, all stem cells (IPSCs, ESCs, and ASCs from the patient) may lose their normal characteristics during handling and *in vitro* expansion, and ultimately transform into a tumorigenic phenotype.

Due to the fact that each small manipulation to cells can potentially increase the chances of mutation, manufacturing stem cells may introduce the unwanted risk of tumor formation (30,31). Generally, the level of stem cell manipulation prior to its clinical application is one of the critical factors relevant to the risk of tumor development. For example, in comparison to ASCs, IPSCs require extensive genetic modification and a reprogramming process. Therefore, the high risk of tumor formation for IPSCs is predictable (32). An additional tumor risk factor associated with reprogrammed stem cells (IPSCs) involves the application of virus vectors such as retroand lentiviruses in order to integrate genes of interest, such as c-MYC, into the stem cell genome. The virus vectors not only increase the potential hazard of oncogene activation but can also reactive one of the reprogramming transgenes, which can subsequently result in tumor formation (33). 

Immune rejection is another pitfall that may occur with stem cell therapy. Due to presentation of host cell markers, autologous transplantation of ASCs evades immune rejection. When the administered stem cells are non-autologous, the immune response may lead to graft rejection (34). Araki et al. (35) have reported that the levels of stem cell differentiation and reprogramming in IPSCs were important factors in the immune response of the patient. T cells in the human immune system can detect residual pluripotency markers such as *OCT4* and eliminate this antigen prior to transplantation, thus avoiding an immune response (36). Although immune suppressive drugs can be used to prevent graft rejection, administration of such drugs can increase the risk of infections and graft maturation inhibition in the patient. Many studies indicate that autologous stem cell transplantations are immuneprivileged and require lower levels of immune suppression (37,39). However, in some cases, by upregulation of major histocompatibility complex (MHC) molecules upon stem cell differentiation, the possibility of immunogenicity increases. In addition, if the administered cells in the recipient are to be used in a non-physiological site or for a nonhomologous function as the donor, the risk of an immune response greatly increases (3). 

In addition to the main stem cell safety issues of tumorigenicity and immunogenicity, several contributed risk factors should be taken into account before the clinical application of stem cells. 

During stem cell cultivation animal sources such as mouse embryonic fibroblast should be used as a feeder layer to support cell growth. These animal sources raise an unwanted risk of passing a number of diseases to humans that are not detectable during screening, especially when strong drugs are administered to wipe out the patient’s immune system. In addition, application of viruses to generate IPSCs can another potential risk that makes the patient immune system vulnerable (40,42). 

Although *in vitro* differentiation is necessary to generate the desired cell type from IPSCs or ESCs prior to transplantation, misdirected growth or dedifferentiation of the administered stem cells is a possible risk that may occur in the patient. Nakanishi have reported that transplanted MS cells from bone marrow for treatment of myocardial infarction resulted in bone characteristics such as ossification and calcification at the injury site. These unexpected dedifferentiation behaviours may have toxic consequences in the patient (43). 

Pharmacokinetic issues such as dosing regimens, administration route and toxicity are also important problems associated with stem cell therapy. The main reason underlying this issue is difficulty in detecting absorption, differentiation and tracking metabolism, and migration of transplanted stem cells in the patient. Therefore, the therapeutic dose and route of administration of stem cells to reach maximal clinical benefit is mostly unclear (26,44). Systemic or local administration of these cells is a matter of debate. It has been shown that systemic administration of MSCs resulted in stem cell accumulation in the lungs, even the administered cells were detected at the injury site. On the other hand, local administration of stem cells and injection of these cells to the myocardia as the site of injury may result in some physiological and anatomical problems such as arrhythmia (45,46). 

Recent studies have attempted to overcome the hurdles of stem cell therapy in order to develop advanced stem cell derivations and enable safe use in humans. A common solution to reduce the tumorigenesis risk and genetic instability of stem cells is the optimization of culture conditions in order to induce genetic stability (47). To achieve this goal, it is crucial to determine the cause of the instability. For example, if oxidative stress is the potential source of instability, changing culture conditions to lower oxygen levels can be helpful (48,24). Exogenous nucleoside supplementation is a useful method if the cause of instability is DNA replicative stress (20,24). One of the potential approaches which can address many aspects of safe stem cell transplantation is genetic modification of stem cells to express suicide genes in the case of tumor development or other negative side effects (49,51). Besides the traditional methods of application of immune suppression drugs and using the patient’s stem cells, DNA methylation or histone modification may likely be helpful to avoid or limit expression of specific MHC or co-stimulatory molecules that lead to reduced susceptibility to rejection (34,52,53). There are some approaches that enhance the post-transplantation safety and efficacy issues of stem cell therapy include integration, maturation, and survival of transplanted cells. These approaches may help to improve use of strategies such as co-transplantation of stem cells with carrier cells, some specific genetic modification to promote the survival and application of anti-inflammatory factors (49,54,55). Although these methods are not 100% successful, it is of benefit to undertake critical evaluations on overcoming tumorigenicity and immunogenicity concerns of stem cells before their use in the clinical setting. 

Despite the advancements in stem cell research technology, a large number of technical and clinical risks remain. Specific concerns exist about the safety of stem cell products and healthy transplantations into humans due to the high potential for tumor formation and immune rejection. Currently, the most extensive studies have determined that tumorigenicity and immunological reactions are the two primary associated risks with stem cell-based therapy, which not only result from extrinsic factors but also depend on the nature of stem cells. Overall, stem cell therapy is a rapidly advancing field that needs to focus more on finding a comprehensive assessment of risk technology and establish systematic follow up monitoring of post-transplantation outcomes. A variety of risk factors (intrinsic and extrinsic) must be considered in order to have safe clinical stem cell therapies. 

Stem cell therapies have a perfect beginning and hold tremendous potential for treatment of multiple challenging diseases. However our understanding is inadequate to apply stem cells as clinical treatments in humans. Stem cell therapy has a number of issues that include ethical, efficacy, side effects, and safety. Ethical considerations can be solved by changing the sources of stem cells, by using ASCs from the patient or IPSC derivation instead of embryonic cells. However, serious biosafety and side effects remain for stem cell therapy, which require further bioanalyses. 
